# Real-time divergent evolution in plants driven by pollinators

**DOI:** 10.1038/ncomms14691

**Published:** 2017-03-14

**Authors:** Daniel D. L. Gervasi, Florian P Schiestl

**Affiliations:** 1 Department of Systematic and Evolutionary Botany, University of Zürich, Zollikerstrasse 107, Zürich 8008, Switzerland

## Abstract

Pollinator-driven diversification is thought to be a major source of floral variation in plants. Our knowledge of this process is, however, limited to indirect assessments of evolutionary changes. Here, we employ experimental evolution with fast cycling *Brassica rapa* plants to demonstrate adaptive evolution driven by different pollinators. Our study shows pollinator-driven divergent selection as well as divergent evolution in plant traits. Plants pollinated by bumblebees evolved taller size and more fragrant flowers with increased ultraviolet reflection. Bumblebees preferred bumblebee-pollinated plants over hoverfly-pollinated plants at the end of the experiment, showing that plants had adapted to the bumblebees' preferences. Plants with hoverfly pollination became shorter, had reduced emission of some floral volatiles, but increased fitness through augmented autonomous self-pollination. Our study demonstrates that changes in pollinator communities can have rapid consequences on the evolution of plant traits and mating system.

## Abstract (web summary)

Pollinators are thought to be a driver of plant diversification, but their effects are difficult to disentangle from those of other biotic and abiotic factors. Here, the authors let plants evolve under different pollination regimes and show rapid and divergent evolution of plant height, floral traits and mating system.

A vast majority of flowering plants are at least partly dependent on animals for pollen transfer^1^; therefore, pollinators play an essential role in ecosystem functioning as well as in human nutrition. However, pollinators are also thought to drive evolutionary diversification of plants. Following the work of pioneering pollination biologists such as Charles Darwin^2^, a modern hypothesis of how pollinators cause floral diversification across a geographic range is the Grant–Stebbins model of pollinator-driven plant divergence^3^,^4^,^5^. In this model, geographical differences in pollinator abundance, the so-called pollinator mosaic, drive adaptive divergence in floral traits across plant populations leading to pollination ecotypes^6^,^7^,^8^,^9^,^10^. A further consequence can be speciation through establishment of reproductive isolation mediated by a lack of pollinator sharing (that is, floral isolation) between incipient plant lineages^11^. A key assumption in this model is that different animal pollinators cause divergent selection on plant traits^12^,^13^,^14^,^15^, the prerequisite for adaptive divergence.

Our understanding of the mechanisms of pollinator-mediated evolution is, however, limited for several reasons. Although many studies have documented selection on floral traits in natural plant populations, they are usually limited in their inference of the mechanism causing a given pattern of selection. This is so because not only pollinators, but also herbivores, pathogens and abiotic factors impose selection on flowers^12^,^16^,^17^. In addition, only a few studies have targeted pollinator-mediated selection in different populations to analyse divergent selection^10^,^18^,^19^,^20^. Studies that document established floral trait differences among plant populations (ecotypes) or species face similar problems in identifying the primary cause for the evolution of such variation, because floral diversification is often linked to shifts in more than one ecological factor, for example in both habitat-type and pollinators^21^,^22^,^23^. As a consequence, 150 years after Darwin's groundbreaking contributions to floral evolution in response to pollinators^2^, we know surprisingly little about how plants respond evolutionarily to changing pollinator environments^24^, which traits evolve first at the onset of adaptation to pollinators and which evolve later, for example, through reinforcement^25^, and what the speed of this process is.

Our study addresses pollinator-mediated divergence employing an experimental approach using a plant with a generalized pollination system. In our experiment, we set up plant lineages originating from the same source population, with lines pollinated either by bumblebees, by hoverflies or by hand during nine consecutive generations, and analysed the resulting evolutionary change. Our approach not only quantifies pollinator-mediated divergent selection, but also evolutionary responses to selection over several generations. Using this novel approach we test the hypothesis, originally outlined in the Grant–Stebbins model, that functionally different pollinators impose divergent selection on plant traits and mating system, leading to the divergence in those traits. Our proof-of-concept study is not only relevant within the context of pollinator-driven diversification, but also to alert us to possible evolutionary consequences of current changes in pollinator communities, including pollinator decline or loss of groups of pollinators^26^,^27^.

Our study shows that plants pollinated by bumblebees evolve taller size and more pronounced floral signals, thereby becoming more attractive to bumblebees. In contrast, hoverflies, being much less efficient pollinators, cause the evolution of spontaneous self-pollination. We conclude that different pollinators lead to diverging evolutionary trajectories in plants, and that the loss of efficient pollinators, such as bees, in natural habitats may have rapid evolutionary consequences for floral traits and mating system in plants.

## Results

### Phenotypic selection

**Figure 1:**
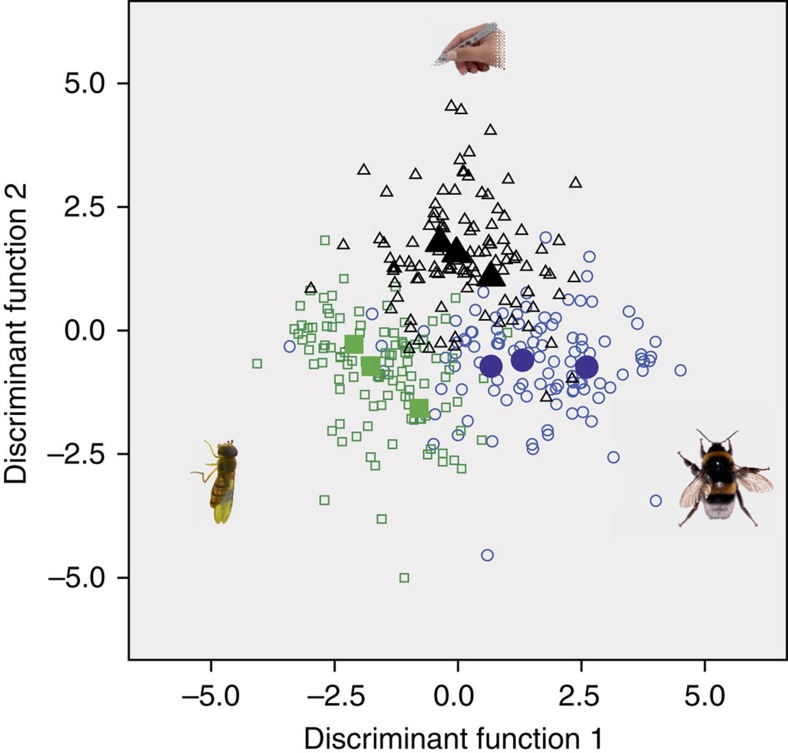
Multivariate comparison of plants after experimental evolution. Plants of nine replicates (total sample size 323) at generation 11 were analysed using linear discriminant function analysis (bumblebee: blue circles, hoverfly: green squares, control hand pollination: black triangles; filled symbols are group centroids of replicates). The analysis comprised morphological traits (petal length and width, flower diameter, pistil length, plant height) and all floral volatiles. In the analysis, only replicates, not treatments were pre-defined. The graph shows that despite floral trait differences among replicates, replicates within treatments resemble each other more than replicates across treatments. The evolved differences are, therefore, better explained by consistent, pollinator-specific selection than by random drift (functions: 1–8 *χ*^2^=1,225.86, 2–8: 881.73, 3–8: 625.16, 4–8: 408.4, 5–8: 248.15, 6–8: 122.12, 7–8: 60.0, all *P*<0.001, 8: 20.32, *P*=0.06). Photos by the authors.

The prerequisite for adaptive evolution is selection. In our experiment, we found that several traits were under significant positive or negative directional selection in bumblebee- and/or hoverfly-pollinated plants (Supplementary Table 1). We also detected significant divergent selection, namely on plant height and the three floral volatiles methyl benzoate, *p*-anisaldehyde and benzyl nitrile (Supplementary Table 1). Few traits were under significant stabilizing or disruptive selection (Supplementary Table 1). To examine whether selection rather than random drift caused our observed evolutionary changes, we analysed trait differences across the replicates of each treatment, looking for repeatable evolutionary patterns. We found such patterns on two levels; first, multivariate analysis showed that overall differences among the replicates of each treatment were largely consistent (Fig. 1). Second, for many individual traits, general linear models in plants of the eleventh generation revealed a significant ‘treatment' effect, indicating differences among treatments were consistent across replicates (Supplementary Table 2; see also ‘Methods' section for justification).

### Evolutionary changes

**Figure 2:**
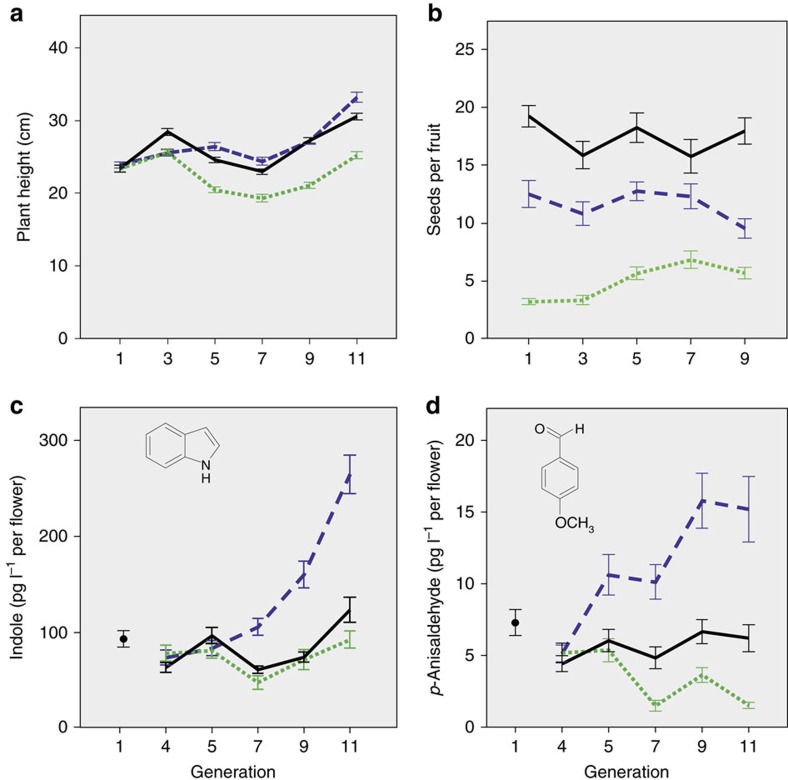
Evolutionary changes in plants throughout experimental evolution. The figure shows mean (±s.e.m.) values per generation in the different pollinator-treatment groups (bumblebees: dashed blue, hoverflies: dotted green, control: solid black; sample sizes are between 82 and 108 per treatment and generation). (**a**) Plant height increased in bumblebee-pollinated plants. (**b**) Pollination efficiency (seeds per fruit) was initially low in hoverfly-pollinated plants but increased throughout the experiment. (**c**) The amount of indole and (**d**) p-anisaldehyde dramatically increased in bumblebee-pollinated plants; *p*-anisaldehyde decreased in hoverfly plants (see also Table 1; Supplementary Table 2).

**Figure 3:**
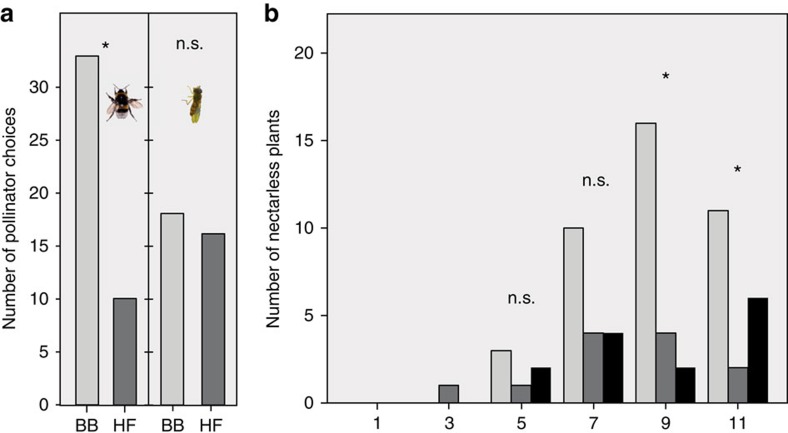
Pollinator preferences and evolution of nectarless plants. (**a**) First choices of bumblebees (left pair of bars, *n*=43) and hoverflies (right pair of bars, *n*=34) when allowed to choose between bumblebee- (BB) and hoverfly-pollinated plants (HF) of generation 11. Bumblebees preferred bumblebee-pollinated plants (binomial test, *P*=0.001), which shows that those plants have adapted to bumblebee preferences. Hoverflies showed no preferences (*P*=0.864). (**b**) Number of nectarless plants in bumblebee- (light grey) hoverfly- (dark grey) and hand-pollinated plants (black) in generations 1–11. The number of nectarless plants was significantly different in generation 9 (generalized linear model with bimodal distribution, *χ*^2^_1_=13.41, *P*=0.001) and 11 (*χ*^2^_1_=6.11, *P*=0.047). Photos by the authors.

In bumblebee-pollinated plants, we detected the most dramatic evolutionary changes in plant size and floral signals. Bumblebee-pollinated plants became taller (Table 1; Fig. 2a), and evolved flowers with larger ultraviolet-reflecting petal area (Table 1), whereas their colour-reflectance spectra remained unchanged (Supplementary Table 3). The total amount of scent emission per flower almost doubled, as more than half of the analysed volatiles showed increased emission (Table 1; Fig. 2c,d). Some of these changes, especially among biosynthetically related volatiles, were not independent, as many of them were correlated with each other (Supplementary Table 4). In dual-choice assays, bumblebees preferred bumblebee-pollinated plants of generation 11 over hoverfly-pollinated plants (Fig. 3a). Despite this elevated attractiveness, bumblebee-pollinated plants showed no augmented fitness over the generations, because efficiency of pollination (that is, seeds per fruit; Fig. 2b) did not increase.

Within plants of all generations, several traits (eight volatiles, flower size and plant height) were correlated with nectar amount, and could thus serve as honest signals (Table 1, Supplementary Tables 2 and 4). On the other hand, nectarless plants became more frequent throughout the experiment. Whereas no nectarless plants were present in the starting generation, the frequency of these (partial) cheaters increased throughout the experiment, becoming most frequent within bumblebee-pollinated plants of generation 9 and 11 (Fig. 3b). Nectarless plants did not differ from nectariferous ones in fitness components and most traits, however, nectarless plants had fewer open flowers, reduced petal width, less methyl benzoate and indole, but more (*Z*)-3-hexenyl acetate and 2-amino benzaldehyde (GLM *P*<0.05).

**Figure 4:**
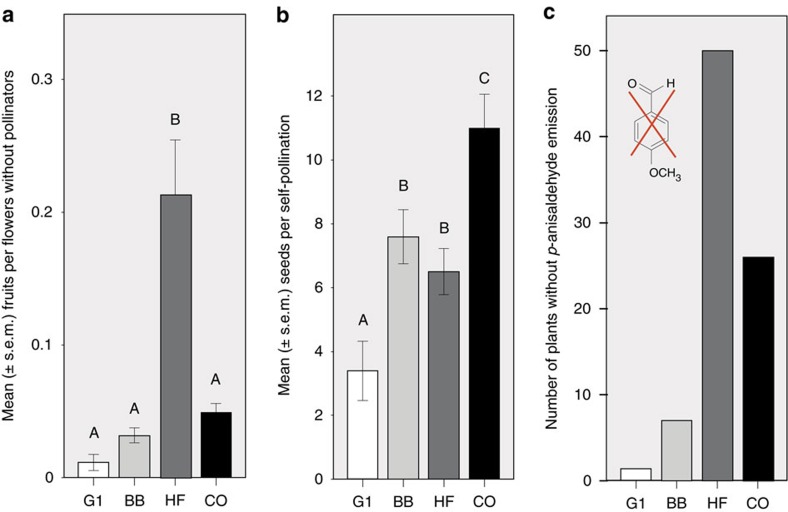
Evolution of mating system and vestigialization in experimental plants. Plants of generation 1 (G1) are compared with the bumblebee-(BB), hoverfly-(HF) and hand-pollinated (CO, control) plants of generation 11. (**a**) Autonomous selfing measured as fruits per flowers produced by plants without access of pollinators (sample sizes: G1: 23, BB: 39, HF: 40, CO: 52). HF-plants evolved increased autonomous selfing (GLM: treatment: F_3,6.04_=11.04, *P*=0.007; different letters indicate differences between groups, LSD *post-hoc* tests, *P*<0.001). Neither ‘seeds per fruit' nor ‘seed weight' were different between the groups (GLM, P>0.05). (**b**) Self-compatibility measured as the ‘number of seeds per self-pollinated flower' (sample sizes: G1: 40, BB: 93, HF: 91, CO: 59). Plants of all pollination groups had elevated self-compatibility in generation 11 (GLM: F_3,6.06_=10.67, *P*=0.008, LSD *post-hoc* tests *P*<0.05). (**c**) Number of plants without *p*-anisaldehyde emission. Frequency of plants without *p*-anisaldehyde was similar among first-generation and eleventh generation BB-plants (*χ*^2^ test, *χ*^2^_1_=2.78, *P*=0.10), but anisaldehyde-loss was more pronounced in CO- and especially in HF-plants, where almost half of all plants had lost the emission of this volatile in generation 11 (G1-BB-HF-CO: *χ*^2^_3_=66.95, *P*<0.001; HF-CO: *χ*^2^_1_=7.58, *P*=0.006).

In hoverfly-pollinated plants, the most significant changes were apparent in mating system. At the start of the experiment, fecundity in these plants was much lower compared to in bumblebee-pollinated plants (GLM, seeds/fruit: F_1,2_=44.47, *P*=0.022; number of fruits: F_1,2_=322.44, *P*=0.003; number of seeds: F_1,2_=220.54, *P*=0.004). These low fitness values, however, increased significantly during the experiment, evidenced by positive regression of fitness components on generation (*P*<0.05 for all replicates in ‘number of seeds' and ‘number of fruits', and in two replicates for ‘seeds per fruit'; Fig. 2b). This increase in fitness was not a consequence of higher pollinator-visitation rates, which were the same in hoverfly- and bumblebee-pollinated plants throughout the experiment (G1: *χ*^2^=0.109, *P*=0.661; G9: *χ*^2^=0.620, *P*=0.431). Hoverfly-pollinated plants, however, showed a 15-fold increase in the ability to produce seeds without pollinators (that is, autonomous selfing; Fig. 4a). Notably, components of inbreeding depression (seed weight and germination rate) did not differ between pollination groups (Supplementary Table 2); self-compatibility, measured as the number of seeds produced by selfed flowers, increased in all treatments (Fig. 4b), likely under selection driven by a limited number of S-alleles being present in our replicate populations.

In correspondence with increased selfing, hoverfly-pollinated plants showed a trend towards reduction in pistil length (Supplementary Table 2; *P*=0.051), and a significant decrease in the emission of the three scent compounds methyl salicylate, *p*-anisaldehyde and indole (Table 1; Supplementary Table 2 and Fig. 4c). One scent compound, benzyl nitrile, increased in hoverfly-pollinated plants (Table 1). In addition, hoverfly-pollinated plants became 1.2 times shorter and flowered later (Table 1; Fig. 2a). Hoverflies showed no preferences for either hoverfly- or bumblebee-pollinated plants of generation 11 (Fig. 3a).

## Discussion

Adaptive evolution is caused by selection on variable and heritable traits in a population. Because many plants are dependent on pollinators for sexual reproduction, pollinators can cause selection and adaptive evolution in traits that maximize their attraction and subsequent pollen delivery^3^,^4^, but this process is difficult to study in nature^24^. Our experiment documents the sole effect of pollinators on adaptive evolution, because all other ecological factors were held constant, and plants derived from the same starting population. We showed that different pollinators can lead to dramatic and rapid divergence, especially in traits that signal to pollinators, as well as in the plants' mating system.

The most dramatic evolutionary changes in the ‘Gestalt' of our experimental plants were the alteration in floral signals and size, likely driven by different preferences of pollinators. Whereas hoverflies are known to have strong innate preferences for yellow flowers^28^, social bees use chemical and visual signals to find rewarding flowers, and their preferences are largely shaped by associative learning^29^,^30^,^31^. Among the multitude of signals emitted by flowers, those that ‘honestly' indicate reward status are thought to be used predominantly^32^. In our experiment, there was a close link between such honest signals and selection mediated by bumblebees, as four of six traits under positive directional selection were correlated with nectar amount, and six of eight traits that increased in bumblebee-pollinated plants showed a correlation with nectar amount. These associations suggest that bumblebees prefer and thus select for honest signals, leading to their evolutionary augmentation.

The amount of nectar reward, however, remained constant in bumblebee-pollinated plants, and the number of nectarless cheaters even increased considerably. These nectarless individuals differed only slightly in their floral signals from nectariferous ones, making it unlikely that bees could have learned to avoid them. Despite the origin of such false-signalling individuals, certain signals in bumblebee-pollinated plants remained honest on a population level. Theoretical models predict a certain proportion of nectarless flowers to be evolutionarily stable in insect-pollinated plant populations^33^, and investigations in natural populations have shown that nectarless flowers are indeed frequently found in many plant species^34^,^35^. Our experiment suggests that bees allow for a greater proportion of nectarless cheaters in a population of otherwise honestly signalling individuals. Perhaps this is because they also collect pollen and consequently do not discriminate much against nectarless flowers if they still produce pollen^36^. Interestingly, among angiosperms, many nectarless species are pollinated by bees, especially if they offer pollen as reward^35^. For hoverflies, nectar seem to be the more important reward, as *E. balteatus* was shown to discriminate against varying sugar concentrations in nectar, but not against different amounts of pollen offered on artificial flowers^37^.

Not unexpectedly, pollinator-mediated selection did not predict all the observed evolutionary changes in our plants. For example, petal length, number of open flowers and methyl benzoate did not increase, despite being under positive selection (but methyl benzoate was under stabilizing selection as well), whereas benzaldehyde and benzyl nitrile did not decrease, despite being under negative directional selection (Supplementary Tables 1 and 2). Several scent compounds increased without being under (detectable) direct selection. These seemingly contradictory findings are likely the consequences of patterns of standing genetic variation or pleiotropies^38^,^39^ (for phenotypic correlations, see Supplementary Table 4), and point out the need for proper quantitative genetics models to infer evolutionary change from phenotypic selection.

In hoverfly-pollinated plants, adaptive evolution took a different trajectory. Those plants did not show any adaptations to the preference of hoverflies, but evolved a massive increase in spontaneous self-pollination. A mix between outcrossing and autonomous selfing is thought to evolve through selection for reproductive assurance under pollen limitation^40^,^41^,^42^,^43^. Pollen limitation is frequently found in natural plant populations, but its cause is often difficult to ascertain^44^. In our experiment, higher pollen limitation in hoverfly-pollinated plants could be concluded to be caused by the lower efficiency of hoverflies in transferring pollen, because plants of all treatment groups originated from the same source population, and ecological conditions as well as insect visitation rates were the same. Whereas a total lack of pollinators has previously been shown to lead to the evolution of self-pollination^4^,^45^, the effects of ‘inferior' pollinators are less clear. Variation in differently-efficient pollinators causing variable pollen limitation is probably common in natural habitats^46^, and may even have increased through human impacts on natural ecosystems^47^.

Our experiments show that pollen limitation can be mitigated by the evolution of autonomous selfing, though at the cost of vestigialization in plant traits. Self-pollination in plants is often associated with a pattern of reduction in floral traits, called the ‘selfing syndrome'^48^,^49^, including smaller flowers that open less widely, less separation between male and female organs, and reduced nectar and scent. We found an evolutionary trend towards the selfing syndrome in hoverfly-pollinated plants in pistil length and some floral scent compounds. Whereas pistil length likely evolves under direct selection to enable autonomous selfing^50^, floral scent reduction is probably the consequence of resource-allocation trade-offs^51^. Other traits such as nectar amount and flower size did not change, likely due to maintained selection by hoverfly pollinators.

In conclusion, our study demonstrates that pollinators are powerful agents of plant evolution, and changes in pollinator communities can have profound and extremely rapid impacts on plant evolutionary trajectories. Thus, altered pollinator environments not only impact ecosystem functioning, but also the evolution of plant traits and mating systems. Among pollinators in decline, bees with their often specific habitat requirements are especially vulnerable^52^,^53^ and seemingly more so than, for example, hoverflies^26^. As a result, pollinator mosaics can shift, with consequences in quantity and quality of pollination^54^, and likely impacts on selection and trait evolution in plants^55^, as shown recently for plant-herbivore^56^ and plant-seed disperser systems^57^. Trait evolution can have downstream effects on plant-pollinator networks and the genetic structure of plant populations^44^, calling for more work on the evolutionary implications of changed pollinator environments in natural habitats.

## Methods

### Experimental design and study system

In 2012, 300 seeds of fast cycling *Brassica rapa* plants (Wisconsin Fast Plants Standard Seed, with high genetic variation) were obtained from Carolina Biological Supplies, and grown in a phytotron under standardized soil, light and watering conditions. These plants are fully outcrossing (self incompatible) and harbour enough standing genetic variation to readily respond to selection^58^,^59^. From these 300 plants, 108 full sib seed families were generated by artificial crossings (only seed families from crosses where both parents produced fruits were used). These 108 full sib seed families were used as the starting population for the experiment.

For the first-generation of the experiment, three treatment groups were established using the 108 families so that each family was represented in each treatment to control for genotype among treatments (Supplementary Fig. 1). Each treatment therefore consisted of 108 plants (representing 108 seed families), which we subdivided into three replicates (A,B,C) each containing 36 plants. The replicates within the treatments were kept as isolated lines during 9 generations (no crosses between replicates were done) to be able to assess independent, repeatable evolutionary changes. The plants of all replicates in all the treatments were grown in the phytotron under standardized soil (Einheitserde classic), light (24 h light) and watering conditions. All plants were phenotyped every second generation starting with generation 1. Floral scent data from generations 1 and 3 were lost due to technical problems; instead, scent was collected from generation 4. Floral scent data of generation 1 were obtained after the end of the experiment by re-growing plants from the starting generation and collecting scent from one plant from each of the 108 seed families. Thus, from the first-generation in total 108 plants (36 from each replicate) were sampled for floral scent at the same time as plants of generation 9.

### Experimental evolution and pollination treatments

In our study we used three pollinator treatments: bumblebees (‘BB', *Bombus terrestris*, Biocontrol, Andermatt, Switzerland), hoverflies (‘HF', *Episyrphus balteatus*, Katz Biotech AG, Germany), and hand pollination. Both insects readily visit flowers of many Brassicaceae species in nature, but represent different functional pollinator categories, and have been shown to vary in abundance in natural habitats^46^. The use of single pollinator species mimics pollinator environments in which the most abundant pollinators are functionally different. In the control treatment (‘CO'), randomly chosen plants were cross-pollinated by hand.

Pollination was performed 23 days after sowing out in a flight cage (2.5 m × 1.8 m × 1.2 m) in the greenhouse under standardized light conditions with bumblebees and hoverflies. Experiments were performed between 0900 hours and 1,500 hours. Bumblebees were held in a separate flight cage in the greenhouse. Hoverflies were purchased as pupae and reared until hatching after which male and female flies were separated. Pollinators were allowed to forage on fast cycling *B. rapa* plants (plants of the control group of the respective generation) and fed with additional pollen until 3 days before the pollination treatment; afterwards, only pollen and sugar solution were provided; 16 h. before pollination, pollinators were starved.

For pollination, all plants of one replicate were randomly placed in a square of 6 × 6 plants with a distance of 20 cm from each other in the flight cage. Five pollinators were added individually and sequentially and each insect was allowed to visit a maximum of three different plants and then removed from the cage; each insect was used only once. In total, 12–15 plants per replicate received one or more visits by pollinators. The overall mean (±s.d.) number of visits (in visited plants) was 1.35±0.63 for bumblebee-pollinated plants and 1.28±0.53 for hoverfly-pollinated plants. For the plants that were visited, the number of visits and number of visited flowers were recorded. In the control group, 12 plants were chosen randomly per replicate and 5 flowers of each plant were hand pollinated by one randomly chosen father plant; fathers were chosen from among the same 12 plants. Each plant could be pollen donor to more than one plant but only received pollen from one plant. After pollination, visited flowers were marked and plants were kept in a cage for additional 30 days until the fruits were collected. Seeds were counted and relative seed set was calculated for each plant by dividing the individual seed set by the mean seed set in the replicate. In addition, number of seeds per fruit was calculated for each visited plant. For each plant male fitness was estimated as predicted paternity (number of pollen export events).

From all seeds produced by the pollinated flowers, a subset of seeds representative of the seed production of each individual was used to grow the next generation. The more seeds a plant produced the more seeds it contributed to the next generation, which again consisted of 36 plants for each replicate. The seed contribution of each visited plant into the next generation was calculated for every replicate as: 36/(replicate sum of seeds/individual seed set). Values below 0.5 were rounded up to 1.

### Inbreeding depression

Inbreeding depression throughout the experiment was quantified by measuring seed weight and germination rate, the latter as percentage of seeds germinated per replicate. To control for trait-changes due to inbreeding depression, seeds produced by plants of the 9th generation were grown (representing the 10th generation) and manually crossed between replicates within the treatments, so that plants of each replicate were pollen donor and pollen recipient for plants of two different replicates (♀A-♂C, ♀B-♂A, ♀C-♂B). Crossings within these combinations of replicates were random. Of the resulting seeds (the eleventh generation) one individual per seed family was grown (36 plants per replicate) under the same conditions as during the experiment. Of these inter-replicate crossings, traits were again measured and used for the final comparison of traits between treatment groups.

### Plant traits

Most traits, including floral scent were measured before pollination, 19–21 days after sowing out. Petal width, length, pistil length and flower diameter of three randomly chosen flowers per plant were measured with an electronic caliper (Digital Caliper 0–150 mm,TOOLCRAFT). Nectar from three flowers was collected with 1 μl micro capillary tubes (Blaubrand, Wertheim, Germany) and the volume determined by measuring the length of nectar column in the micropipette with a caliper. For the quantification, the mean of three flowers was used. For 157 plants evenly split across the treatments, the sugar content of the nectar was determined using derivatization and gas chromatographic analysis. To do so, nectar was transmitted to filter paper stored in silica gel. The sector on the filter paper containing the nectar was cut from the rest of the filter paper and nectar was eluted in 1 ml high-purity Mili-Q water by shaking the dilution for 90 min with 400 r.p.m. at 60 °C on a laboratory shaker. Afterward 50 μl of the solution were dried at 60 °C and derivatized with 100 μl of a mixture of anhydrous pyridine (Fisher Scientific, Geel, Belgium), hexamethylsilazane (Sigma-Aldrich, Buchs, Switzerland) and trimethylchlorosilane (Sigma-Aldrich, Buchs, Switzerland) (10:5:3). Subsequently, samples were run by GC–MS as described in ref. 32. We calculated total sugar amounts per flower and inflorescence as the sum of all different sugars (fructose, glucose, sucrose and sorbitol). The correlation between nectar sugar content and nectar volume was positive and high (*r*_156_=0.732, *P*<0.001), thus, for the remaining plants, only nectar volume was determined. Floral scent collection was done before bioassays in a nondestructive way from all plant inflorescences as soon as at least five flowers were open. We used headspace sorption with a push-pull system^59^,^60^. The inflorescences of the plants were enclosed in glass cylinders previously coated with sigmacote (Sigma-Aldrich) and closed with a Teflon plate. The number of open flowers was counted for each plant. Air from the surrounding was pushed with a flow rate of 100 ml min^−1^ trough activated charcoal filters into the glass cylinder. Simultaneously, air was pulled from the glass cylinder with a flow rate of 150 ml min^−1^ trough a glass tube filled with ∼30 mg Tenax TA (60/80 mesh; Supleco, Bellefonte, PA, USA). Air from empty glass cylinders was collected as air controls. Floral volatiles were collected for two hours in a phytotron under standardized light and temperature conditions. Quantification of volatiles was conducted by gas chromatography with mass selective detection (GC–MSD). Samples were injected into a GC (Agilent 6890N; Agilent Technologies, Palo Alto, CA, USA) by a MultiPurpose Sampler (MPS; Gerstel, Müllheim, Germany) using a Gerstel thermal desorption unit (TDU; Gerstel) with a cold injection system (CIS; Gerstel). For thermodesorption, the TDU was heated from 30 to 240 °C at a rate of 60 °C min^−1^ and held at a final temperature for 1 min. The CIS was set to −150 °C during the trapping of eluting compounds from the TDU. For injection, the CIS was heated to 250 °C at a rate of 12 °C s^−1^, and the final temperature was held for 3 min. The GC was equipped with a HP-5 column (0.25 mm diameter, 0.25 μm film thickness, 15 m length), and helium was used as carrier gas at a flow rate of 2 ml min^−1^. Compound identification and quantification were done following^60^ with the Agilent MSD ChemStation Program. Quantification of compounds was obtained through measurement of peak areas of selected target ions specific to the individual scent compounds. Specific target ions were obtained from synthetic standards of all compounds; peak areas were converted into absolute amounts using calibration curves previously obtained for each compound using synthetic compounds in three different concentrations. Only scent compounds that were present in significantly higher amounts than in the air control were included in the analysis (in total 14 scent compounds). All amounts of volatiles were calculated in pg per flower l^−1^ sampled air.

Twenty-three days after sowing out, on the same day as pollination was done, the number of open flowers and height of each plant were recorded. After pollination (but on the same day) the colour-reflectance spectra of three petals from different unpollinated (when possible) flowers per plant were recorded using a fiberoptic spectrophotometer (AvaSpec-2048; Avantes, Apeldoorn, the Netherlands) and a Xenon pulsed light source (AvaLight-XE; Avantes). One petal at a time was placed under the spectrophotometer (specifically focusing on the distal part of the petal) and the percentage reflectance (relative to a white standard) between 200 and 900 nm every 0.6 nm was recorded in transmission mode. Of the spectrum measured, only the mean of the reflectance values every 10 nm from 260 to 650 nm from the three petals were used in the analysis. In plants of the eleventh generation, a subset of ca 20 plants per replicate was analysed for colour, because none of the colour PCs was found to be under selection throughout the experiment. The area of the ultraviolet absorbing and reflecting petal surface was measured only in plant of generation 11 with a ultraviolet-sensitive digital camera with quartz lens. Pictures of flowers were taken and ultraviolet absorbing area quantified using the software package ImageJ ( https://imagej.nih.gov/ij/).

### Pollinator preference assays

Assays for pollinator preferences were conducted for each replicate with both types of pollinators. For each replicate, two behavioural assays were performed (one for each pollinator-treatment). Bumblebee- and hoverfly-pollinated plants (generation 11) of each replicate were randomly paired and placed side-by-side (ca 30 cm distance) in a flight cage (2.5 m × 1.8 m × 1.2 m). One pollinator was placed into the cage and allowed to visit one plant. Pollinators were immediately caught after they made their choice. Each plant-pair was assayed with one pollinator.

### Self-compatibility and autonomous selfing

To test for self-compatibility, we grew plants from the first (15 plants per replicate) and the eleventh generation (30 plants per replicate). One seed per seed family (from randomly chosen families) was grown and two flowers per plant selfed at anthesis. The mean number of seeds produced per selfed flower for each individual plant was used as a measurement of self-compatibility.

To test for autonomous selfing, we grew ca 12 plants (one seed per family) per replicate from every treatment of generation 11 and 1 (in total 162 plants). After 30 days when ca 20 flowers had opened, the remaining buds in each plant were carefully cut and the number of opened flowers was recorded. The plant was then allowed to develop fruits without any insects accessing the plants. After ripening of the fruits, seeds were collected and number of seeds was counted and weighed for each plant. The number of fruits per open flower and seed per fruits were used as a measurement for autonomous selfing. Because a few plants had a very high number of fruits per open flowers, we deleted these outliers for the final comparison of autonomous selfing. The following number of outliers were deleted: 1 in generation 1; in G11: 2 in BB, 3 in HF, 2 in CO.

### Statistical analysis

To analyse phenotypic selection, selection differentials and gradients were calculated through regressing plant fitness onto traits^61^. This analysis was done separately for the treatments, but for all replicates and generations combined. As a fitness estimate, ‘number of visits' was used, which was a counting variable and followed a Poisson distribution. Another fitness variable, ‘relative seed set' had a distibution biased by the many zero values; in addition, seed set missed the only male fitness component of the first plant being visited, which did not set seed from this visit (because pollinators initially did not carry *Brassica* pollen). The number of visits was, however, strongly correlated with relative seed set (BB: *r*_626_=0.694, *P*<0.001; HF: *r*_605_=0.597, *P*<0.001). Generalized linear models (with Poisson distribution) were used to calculate selection gradients (multivariate) and differentials (univariate) for every treatment with number of visits as dependent variable and traits as covariates. In addition, quadratic selection gradients were calculated with all traits and the squared term of each trait added to the model, and subsequently gradients doubled^62^. To check for differences in selection between bumblebees and hoverflies, a generalized linear model (with Poisson distribution) with number of visits as dependent variable, treatment as fixed factor, plant traits as covariates and the interaction treatment*plant trait was performed. Before the selection analysis, all variables were standardized to mean=0 and s.d.=1 (*Z*-values) at the replicate level. A generalized linear model was also used to compare visitation rates between bumblebee- and hoverfly-pollinated plants across all generations. Floral colour spectrophotometer values were reduced through principal component (PC) analysis with varimax rotation. Only PCs with an eigenvalue above one were used in the analysis.

Evolutionary change in plants traits was assessed in plants of the 11th generation using multivariate linear discriminant function analysis and univariate general linear models (GLM). For GLM, each trait was used as the dependent variable, replicate as random factor and treatment as fixed factor with LSD *post-hoc* test. To discriminate the impact of natural selection from drift, we assessed whether trait differences were consistent among replicates of a given pollination treatment. In the GLM analysis, a significant ‘treatment' effect indicates trait difference between different pollinator groups across all replicates, and thus discriminates pollinator-specific evolution from drift. Drift would be indicated by evolutionary changes in some (random) replicates only, indicated by a significance in the factor ‘replicate' or interaction between ‘replicate' and ‘treatment'. Self-compatibility and autonomous selfing were assessed by GLM, too, but values of first-generation plants were included in the analysis. For the analyses of volatiles and nectar volume, data were ln(1+*x*) transformed to approach normal distribution. For the GLM with the colour variables, a PC analysis was performed as described above but without prior standardization of the variables. The PC analysis was performed for all treatments, replicates and all generations together resulting in four PCs explaining 96.9% of the total variance. The frequency of nectarless flowers was analysed separately for each generation, by using generalized linear models with bimodal distribution, with ‘presence of nectar' (yes/no) as the dependent variable, and treatment and replicate as factors. Traits in nectariferous and nectarless flowers were compared for the ninth and eleventh generation together, by using general linear models with the trait as the dependent variable, and ‘presence of nectar' and treatment as fixed factors. The first choice preferences of bumblebees and hoverflies were analysed by binomial test (test-prop=0.5; all replicates pooled). Correlations between nectar and plant traits were calculated for all generations combined using Pearson product-moment correlations with ln-transformed values. Statistics were performed with IMB SPSS Statistics (Version 20.0.0, http://www-01.ibm.com/software/analytics/spss/products/statistics/).

### Data availability

Data are available upon request from the corresponding author.

## 

## Additional information

**How to cite this article:** Gervasi, D.D.L. & Schiestl, F. P. Real-time divergent evolution in plants driven by pollinators. *Nat. Commun.* **8,** 14691 doi: 10.1038/ncomms14691 (2017).

**Publisher's note:** Springer Nature remains neutral with regard to jurisdictional claims in published maps and institutional affiliations.

## Supplementary Material

Supplementary Information Supplementary Figures and Supplementary Tables

## Figures and Tables

**Table 1:** Traits that evolved differences between plants during the experiment.

**Trait**	**Correlation (nectar)**	**Bumblebee**	**Hoverfly**	**Control**
Plant height (cm)	**0.07**	33.18±7.32^A^	25.15±5.25^B^	30.63±4.43^C^
Days to flowering	**−0.17**	17.68±1.14^A^	18.65±1.02^B^	17.67±0.82^A^
Ultraviolet-reflecting area (%)	n.a.	49.78±6.35^A^	42.48±5.05^B^	41.64±8.31^B^
(*E*,*E*)-α-farnesene	**0.12**	1,039.77±541.58^A^	919.78±401.59^AB^	849.31±448.50^B^
Phenylacetaldehyde	**0.15**	233.40±296.83^A^	40.31±54.64^B^	46.78±75.94^B^
Phenylethyl alcohol	0.04	7.72±10.42^A^	1.70±2.43^B^	1.94±2.44^B^
Methyl salicylate	−0.01	35.58±36.75^A^	14.91±13.17^B^	52.18±52.72^C^
*p*-Anisaldehyde	−0.02	15.20±23.53^A^	1.52±2.29^B^	6.20±9.76^C^
Benzyl nitrile	**0.15**	130.63±80.66^A^	56.56±49.25^B^	46.92±54.97^C^
Indole	**0.11**	264.81±206.39^A^	92.56±91.93^B^	123.63±134.78^C^
Methyl anthranilate	**0.05**	585.48±459.99^A^	209.24±186.68^B^	251.88±287.58^B^
Total volatile emission	n.a.	4,111.18±2,015.09^A^	2,286.43±1,044.97^B^	2,409.57±1,506.37^B^

Mean (±s.d.) trait values of plants of different pollinator treatments in generation 11 are shown. For all these traits, the factor ‘treatment' in the GLM analysis was significant (*P*<0.05). Different superscript letters (A, B, C) indicate significant differences between treatment groups assessed with LSD *post-hoc* tests. ‘Correlation (nectar)' gives Pearson correlation coefficients of each trait with ‘nectar per flower', calculated for plants of all treatments and all generations together (values in bold: *P*<0.05; n.a.: not analysed). Values for volatiles are in pg l^−1 ^h^−1 ^flower^−1^. Sample sizes are between 106 and 109 for all traits, except for ultraviolet area, between 45 and 50. See Supplementary Table 2 for all traits and detailed statistical values.
