# Supplementary material for: Real-time divergent evolution in plants driven by pollinators
Source: Nat Commun. 2017 Mar 14;8:14691. doi: 10.1038/ncomms14691 (PMC5424062; doi:10.1038/ncomms14691)
Supplement: Supplementary Information — Supplementary Figures and Supplementary Tables [file ncomms14691-s1.pdf]

Supplementary Information

Daniel Gervasi, Florian P Schiestl

Real time divergent evolution in plants driven by pollinators

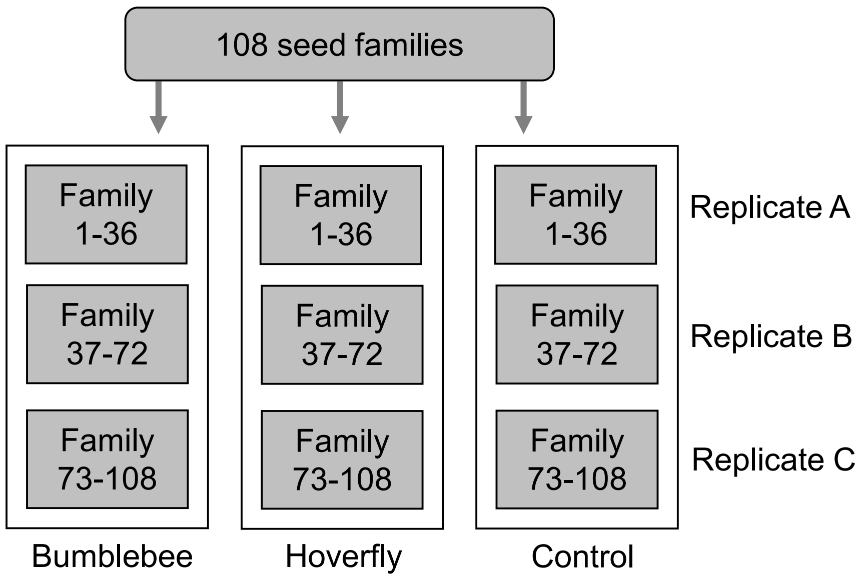

Supplementary Figure 1 Design of our evolution experiment at generation one. 108 full sib seed families of fast cycling *B. rapa* plants were split into three treatment groups and three replicates therein. Each replicate consisted of 36 plants. Families were replicated across treatments, to control for genotype among treatment groups.

Supplementary Table 1 Linear (ß) and quadratic (γ) selection gradients and differentials (*S*) (± standard err) on all measured traits in the different treatment groups. Significant selection coefficients (P<0.05) are given in bold. Asterisks indicate significantly different linear selection gradients between bumblebee- and hoverfly- plants (GLM P<0.05).

**Trait**	**Bumblebee**			**Hoverfly**			**Control**		
	**ß**	**γ**	***S***	**ß**	**γ**	***S***	**ß**	**γ**	***S***
Plant height	**0.353±0.077***	0.006±0.050	**0.562±0.064**	0.070±0.075	-0.133±0.056	**0.198±0.064**	0.029±0.086	-0.041±0.064	0.094±0.077
No. open flowers	**0.390±0.067**	-0.127±0.048	**0.503±0.057**	**0.209±0.067**	**-0.206±0.049**	**0.268±0.058**	**0.147±0.076**	**-0.286±0.068**	**0.161±0.072**
Pistil length	-0.072±0.072	-0.040±0.057	0.083±0.064	-0.006±0.067	-0.020±0.051	0.034±0.064	0.001±0.081	-0.034±0.059	0.053±0.076
Petal length	**0.260±0.103**	-0.161±0.062	**0.215±0.064**	0.063±0.102	-0.178±0.065	**0.173±0.065**	0.172±0.108	-0.135±0.068	**0.164±0.075**
Petal width	0.159±0.085*	-0.066±0.060	**0.325±0.065**	-0.100±0.075	-0.080±0.053	0.037±0.064	-0.038±0.092	-0.092±0.069	0.068±0.076
Flower diameter	-0.116±0.107	0.181±0.050	**0.159±0.065**	0.144±0.104	0.226±0.065	**0.208±0.067**	-0.008±0.106	0.051±0.062	0.116±0.078
Nectar per flower	0.013±0.070	0.002±0.038	0.120±0.069	0.094±0.072	-0.024±0.035	0.125±0.070	0.090±0.083	-0.007±0.052	0.097±0.081
ß-Pinene	0.142±0.075	-0.098±0.055	0.070±0.069	-0.075±0.088	0.097±0.048	-0.115±0.077	0.034±0.092	-0.117±0.063	0.044±0.084
(*E,E*)-α-Farnesene	-0.028±0.087	0.000±0.049	0.066±0.072	0.113±0.092	0.137±0.060	0.031±0.075	**0.277±0.105**	-0.048±0.070	**0.264±0.091**
Benzaldehyde	**-0.211±0.089**	-0.177±0.062	-0.027±0.070	-0.102±0.98	-0.255±0.070	-0.121±0.074	-0.064±0.102	-0.068±0.062	0.011±0.085
Phenylacetaldehyde	0.233±0.124	0.248±0.070	0.100±0.068	-0.071±0.121	0.247±0.081	-0.074±0.076	0.003±0.150	0.072±0.090	0.073±0.083
Phenylethyl alcohol	-0.140±0.112	-0.017±0.048	0.005±0.070	-0.027±0.112	-0.042±0.073	-0.087±0.076	0.018±0.132	-0.082±0.062	0.082±0.084
Methyl benzoate	**0.322±0.105***	**-0.312±0.079**	**0.257±0.071**	-0.071±0.112	0.096±0.078	-0.065±0.075	0.085±0.109	-0.044±0.062	0.072±0.085
Methyl salicylate	-0.028±0.095	0.195±0.076	0.085±0.071	0.072±0.091	-0.117±0.070	0.004±0.075	-0.064±0.102	0.234±0.064	-0.006±0.085
p-Anisaldehyde	**0.211±0.088***	0.108±0.061	**0.166±0.069**	-0.069±0.090	0.161±0.060	-0.033±0.075	-0.057±0.103	-0.029±0.074	0.000±0.085

Trait	Bumblebee			Hoverfly			Control		
	**ß**	**γ**	***S***	**ß**	**γ**	***S***	**ß**	**γ**	***S***
Benzyl nitrile	**-0.392±0.145***	0.025±0.081	0.035±0.070	0.108±0.115	0.134±0.061	-0.001±0.075	-0.066±0.167	0.247±0.091	0.078±0.085
2-Amino benzaldehyde	0.105±0.117	0.046±0.075	0.063±0.072	0.027±0.111	0.153±0.071	0.089±0.077	0.178±0.151	-0.088±0.095	0.120±0.089
Indole	**0.297±0.135**	-0.217±0.090	**0.174±0.072**	0.106±0.119	**-0.481±0.090**	0.034±0.075	-0.059±0.144	-0.157±0.088	0.098±0.087
Methyl anthranilate	-0.071±0.126	-0.197±0.082	**0.182±0.076**	-0.174±0.123	-0.292±0.087	-0.036±0.074	-0.014±0.127	0.043±0.076	0.083±0.086
Z-(3)-Hexenyl acetate	-0.024±0.080	-0.109±0.065	-0.034±0.070	-0.156±0.095	-0.005±0.059	**-0.192±0.081**	-0.022±0.095	-0.210±0.085	0.019±0.084
1-Butene-4-isothyocyanate	-0.116±0.076	0.057±0.056	**-0.145±0.072**	0.004±0.082	-0.194±0.066	-0.055±0.076	0.077±0.089	-0.059±0.068	0.089±0.083
PC1 color (500-650nm)	-0.063±0.063	-0.027±0.041	-0.056±0.062	0.061±0.022	0.061±0.044	0.061±0.063	0.097±0.075	-0.029±0.051	0.100±0.075
PC2 color (290-400nm)	0.067±0.065	0.068±0.052	0.063±0.063	0.014±0.064	0.016±0.049	0.005±0.064	-0.112±0.074	-0.034±0.058	-0.114±0.074
PC3 color (400-500nm)	-0.064±0.064	-0.164±0.049	-0.065±0.064	0.037±0.061	-0.135±0.046	0.033±0.061	-0.004±0.076	-0.075±0.053	-0.001±0.077
PC4 color (260-290nm)	0.037±0.061	-0.039±0.044	0.029±0.060	-0.106±0.067	-0.093±0.051	-0.106±0.066	-0.024±0.076	0.016±0.045	-0.030±0.076

Supplementary Table 2 Trait differences (mean ± s.d.) between plants of the different treatment groups at generation 11 (after interreplicate crossings) and correlations with floral nectar. "Corr." gives Pearson correlation coefficients of each trait with "nectar per flower", calculated for plants of all treatments and all generations together (values in bold have P<0.05). Values for traits are in cm for morphological traits and $pg*l^{-1}*hr^{-1}*flower^{-1}$ for scent variables if not otherwise indicated. Variables with significant differences between treatment groups are given in bold; different superscript letters (A, B, C) indicate significant differences between individual groups. For statistical analysis, a general linear model with "treatment" as fixed and "replicate" as random factor and LSD post hoc test was performed. All scent and nectar variables and were logarithmically transformed before statistical analysis.

Trait	Corr.	Bumblebee	Hoverfly	Control	Source	Df	F	P
**Plant height**	**0.07**	**33.18±7.32^{A}**	**25.15±5.25^{B}**	**30.63±4.43^{C}**	**Treatment**	2,4	**15.37**	**0.013**
					**Replicate**	2,4	**10.41**	**0.026**
					**T*R**	4,318	**4.52**	**0.001**
No. open flowers	0.02	9.83±2.65	10.01±2.70	9.17±2.27	Treatment	2,4	0.86	0.489
					Replicate	2,4	3.38	0.138
					**T*R**	4,317	**4.37**	**0.002**
**Days to flowering**	**-0.17**	**17.68±1.14^{A}**	**18.65±1.02^{B}**	**17.67±0.82^{A}**	**Treatment**	2,4	**10.92**	**0.024**
					Replicate	2,4.4	3.21	0.138
					**T*R**	4,428	**4.09**	**0.003**
Pistil length	-0.03	0.92±0.11	0.83±0.13	0.94±0.10	Treatment	2,4	6.85	0.051
					Replicate	2,4	3.42	0.136
					T*R	4,125	1.99	0.099
Long stamen length	n.a.	0.72±0.10	0.65±0.13	0.75±0.07	Treatment	2,4	3.09	0.154
					Replicate	2,4	1.62	0.305
					**T*R**	4,125	**4.57**	**0.002**
Short stamen length	n.a.	0.59±0.08	0.51±0.11	0.58±0.09	Treatment	2,4	1.65	0.300
					Replicate	2,4	1.19	0.392
					**T*R**	4,121	**6.317**	**<0.001**
Petal length	**0.05**	0.56±0.06	0.54±0.05	0.57±0.05	Treatment	2,4	1.95	0.256
					Replicate	2,4	0.14	0.872
					**T*R**	4,318	**4.35**	**0.002**

Trait	Corr.	Bumblebee	Hoverfly	Control	Source	Df	F	P
Petal width	0.04	0.51±0.06	0.46±0.05	0.44±0.05	Treatment	2,4	4.83	0.086
					Replicate	2,4	2.23	0.223
					**T*R**	4,318	**10.16**	**<0.001**
Flower diameter	**0.13**	1.35±0.11	1.30±0.10	1.34±0.12	Treatment	2,4	2.86	0.169
					Replicate	2,4	0.35	0.721
					**T*R**	4,318	**2.86**	**0.024**
**UV reflecting area (%)**	n.a.	**49.78±6.35**^{A}	**42.48±5.05**^{B}	**41.64±8.31**^{B}	**Treatment**	2,4.03	**40.97**	**0.002**
					**Replicate**	2, 4.04	**7.23**	**0.046**
					T*R	4,130	0.5	0.739
Nectar per flower (nl)		358.21±388.63	338.28±335.00	255.58±239.72	Treatment	2,4	1.36	0.354
					Replicate	2,4	0.13	0.885
					T*R	4,318	2.18	0.074
ß-Pinene	0.03	4.82±4.29	5.09±4.54	4.34±2.39	Treatment	2,4	0.09	0.913
					Replicate	2,4	2.74	0.178
					**T*R**	4,314	**6.25**	**<0.001**
**(*E,E*)-α-Farnesene**	**0.12**	**1039.77±541.58**^{A}	**919.78±401.59**^{AB}	**849.31±448.50**^{B}	**Treatment**	2,4	**7.76**	**0.042**
					**Replicate**	2,4	**7.98**	**0.040**
					T*R	4,314	0.54	0.704
Benzaldehyde	**0.08**	289.06±161.02	381.22±264.43	259.36±147.20	Treatment	2,4	1.93	0.259
					Replicate	2,4	0.11	0.110
					**T*R**	4,314	**3.56**	**0.007**
**Phenylacet-aldehyde**	**0.15**	**233.40±296.83**^{A}	**40.31±54.64**^{B}	**46.78±75.94**^{B}	**Treatment**	2,4	**32.05**	**0.003**
					Replicate	2,4	2.87	0.169
					**T*R**	4,314	**2.82**	**0.025**
**Phenylethyl alcohol**	0.04	**7.72±10.42**^{A}	**1.70±2.43**^{B}	**1.94±2.44**^{B}	**Treatment**	2,4	**27.26**	**0.005**
					Replicate	2,4	0.64	0.573
					**T*R**	4,314	**2.75**	**0.028**
Methyl benzoate	**0.23**	163.02±152.00	109.51±104.52	84.47±71.03	Treatment	2,4	1.49	0.328
					Replicate	2,4	1.89	0.265
					**T*R**	4,314	**7.33**	**<0.001**
**Methyl salicylate**	-0.01	**35.58±36.75**^{A}	**14.91±13.17**^{B}	**52.18±52.72**^{C}	**Treatment**	2,4	**17.67**	**0.010**
					**Replicate**	2,4	**10.12**	**0.027**
					T*R	4,314	1.41	0.231

Trait	Corr.	Bumblebee	Hoverfly	Control	Source	Df	F	P
**p-Anisaldehyde**	-0.02	**15.20±23.53^{A}**	**1.52±2.29^{B}**	**6.20±9.76^{C}**	**Treatment**	2,4	**15.31**	**0.013**
					Replicate	2,4	1.09	0.419
					**T*R**	4,314	**4.43**	**0.002**
**Benzyl nitrile**	**0.15**	**130.63±80.66^{A}**	**56.56±49.25^{B}**	**46.92±54.97^{C}**	**Treatment**	2,4	**16.02**	**0.012**
					Replicate	2,4	0.26	0.783
					**T*R**	4,314	**3.92**	**0.004**
2-Amino. benzaldehyde	**0.14**	1194.17±876.50	361.62±377.91	557.79±645.36	Treatment	2,4	4.70	0.089
					Replicate	2,4	0.73	0.536
					**T*R**	4,314	**8.34**	**<0.001**
**Indole**	**0.11**	**264.81±206.39^{A}**	**92.56±91.93^{B}**	**123.63±134.78^{C}**	**Treatment**	2,4	**12.05**	**0.020**
					Replicate	2,4	0.49	0.643
					**T*R**	4,314	**4.22**	**0.002**
**Methyl anthranilate**	**0.06**	**585.48±459.99^{A}**	**209.24±186.68^{B}**	**251.88±287.58^{B}**	**Treatment**	2,4	**18.82**	**0.009**
					**Replicate**	2,4	**11.05**	**0.023**
					T*R	4,314	0.91	0.456
Z-(3)-Hexenyl acetate	-0.03	73.15±63.60	34.71±43.88	85.95±117.78	Treatment	2,4	1.97	0.254
					Replicate	2,4	0.86	0.490
					**T*R**	4,314	**11.68**	**<0.001**
1-Butene-4-isothiocyate	0.05	47.47±46.89	57.69±69.42	38.81±39.06	Treatment	2,4	0.16	0.861
					Replicate	2,4	3.82	0.118
					**T*R**	4,314	**7.68**	**<0.001**
**Total volatile emission**	n.a.	**4111.18±2015.09^{A}**	**2286.43±1044.97^{B}**	**2409.57±1506.37^{B}**	**Treatment**	2,4	**14.44**	**0.015**
					Replicate	2,4	0.29	0.765
					**T*R**	4,314	**3.26**	**0.012**
Seed weight[1] (mg)	n.a.	1.46±0.47	1.52±0.70	1.52±0.54	Treatment	2,4	0.24	0.799
					**Replicate**	2,4	**11.04**	**0.023**
					**T*R**	4,97	**6.62**	**<0.001**
Germination rate (%)[1,2]	n.a.	0.82±0.28	0.92±0.06	0.98±0.03	Treatment	2	0.71	0.527

[1]Measured in 9th generation; [2]assessed on replicate level; n.a.: not analyzed

Supplementary Table 3 Analysis of color spectrophotometer vales (principle components) for plants of generation 11 using general linear models.

**Color variable**	**source**	**DF**	**F**	**P**
Color reflectance PC1	Treatment	2,4	1.9	0.262
(500-650nm)	Replicate	2,4.6	3.88	0.102
	T*R	4,172	0.59	0.670
Color reflectance PC2	Treatment	2,4	0.07	0.932
(290-400nm)	Replicate	2,4	0.97	0.483
	**T*R**	**4,172**	**5.67**	**<0.001**
Color reflectance PC3	Treatment	2,4	1.27	0.374
(400-500nm)	Replicate	2,4.1	0.23	0.805
	T*R	4,172	3.84	0.005
Color reflectance PC4	Treatment	2,4	1.32	0.363
(260-290 nm)	Replicate	2,4	3.90	0.114
	**T*R**	**4,172**	**8.94**	**<0.001**

Supplementary Table 4 Pearson product-moment correlation heatmap using plants of all generations and treatments combined (n=1894 for morphology-morphology, 1654 scent-scent, 1244 morphology-scent, 919 color-scent, 1563 color-morphology; nectar is included in morphology). Nectar and all scent variables were ln 1+x transformed. Positive correlations are given in shades of red, negative correlation in shades of blue. Correlation coefficients are shown inside the cells. *P<0.05, **P<0.01.

	Plant height	No. of open fl..	Pistil length	Petal length	Petal width	Flower diameter	Nectar	ß-Pinene	(*E*)-α-Farnesene	Benzaldehyde	Phenylacetaldehyde.	Phenyl ethyl alcohol	Methyl benzoate.	Methyl salicylate	p-Anisaldehyde	Benzyl nitrile	2-Aminobenzaldehyde	Indole	Methyl anthranilate.	(*Z*)-3-Hexenyl acetate	1-Butene-4-isothiocyanate	Color PC1	Color PC2	Color PC3
No. op. flo.	.354**																							
Pist. length	.232**	.041																						
Pt. length	.105**	.057*	.385**																					
Pt. width	.171**	.065**	.193**	.467**																				
Fl. diameter	.176**	.057*	.303**	.739**	.481**																			
Nectar	.074**	.022	-.031	.047*	.041	.125**																		
ß-Pinene	.032	-.179**	-.095**	-.168**	-.138**	-.049	.027																	
E-α-Farn.	.220**	.011	-.050	-.030	-.008	.031	.115**	.225**																
Benzald.	.026	-.083**	.001	.022	-.020	.069*	.078**	.395**	.255**															
Phenylac.	.241**	-.071*	.125**	-.024	.092**	.099**	.146**	.181**	.250**	.213**														
Phenyleth.	.134**	.052	.185**	.104**	.038	.092**	.039	.150**	.249**	.358**	.650**													
Meth.benz.	.264**	.098**	.109**	-.056*	-.017	.022	.226**	.214**	.273**	.375**	.309**	.304**												
Meth.sal.	.111**	-.023	.160**	.099**	.056*	.055	-.012	.088**	.136**	.154**	.145**	.250**	.474**											
pAnisald.	.190**	.040	.060*	.096**	.212**	.123**	-.016	.070**	.122**	.257**	.290**	.327**	.050*	.211**										
Benzylnitr.	.151**	-.061*	.067*	.033	.098**	.124**	.148**	.204**	.255**	.284**	.676**	.530**	.358**	.234**	.402**									
Aminoben.	.264**	-.085**	.019	.065*	.194**	.194**	.141**	.056*	.196**	.193**	.370**	.227**	-.072**	.094**	.450**	.506**								
Indole	.310**	-.031	.009	.035	.176**	.134**	.109**	.104**	.311**	.225**	.466**	.365**	.269**	.201**	.389**	.652**	.653**							
Methanth.	.301**	.102**	.062*	-.010	.128**	.064*	.055*	.090**	.262**	.121**	.335**	.337**	.594**	.512**	.268**	.514**	.303**	.575**						
*Z*3hex.ac.	.168**	-.199**	.035	-.047	-.024	-.015	-.034	.290**	.222**	.255**	.107**	.184**	.157**	.217**	.072**	.109**	.012	.124**	.113**					
But.isothio.	.057*	-.053	-.073*	-.161**	-.159**	-.125**	.045	.286**	.225**	.301**	.031	.184**	.254**	.049*	-.027	.101**	-.035	.107**	.139**	.319**				
Color PC1	-.010	-.020	-.013	.101**	.106**	.109**	.052*	.037	.065*	.030	.033	.044	.007	.012	.007	.074*	.028	.060	.012	-.034	-.014			
Color PC2	.027	.012	.018	-.010	-.027	-.029	-.026	.000	.001	-.005	.005	-.030	-.016	-.001	.031	-.058	-.065	-.070*	-.026	-.003	.069*	.000		
Color PC3	.010	.126**	-.051*	.028	-.002	.027	.023	-.068*	.025	-.052	.002	-.007	.005	-.087**	.019	-.033	.000	.018	-.055	-.046	-.014	.000	.000	
Color PC4	.042	.011	.037	.006	.039	-.022	.004	.064	-.039	.014	.020	.020	-.029	-.050	-.005	-.002	.024	-.027	-.048	.040	.034	.000	.000	.000
